# Round the Hospitals

**Published:** 1919-07-19

**Authors:** 


### ROUND THE HOSPITALS.

The King held an Investiture at Buckingham Palace on July 10, and personally conferred decorations on members of the Nursing Services as follows: The Royal Red Cross, First Class: Sister Agnes Taylor, Q.A.I.M.N.S.R.; Mrs. Jessie Laurie, B.R.C.S.; Miss Emma Pense, Canadian A.N.S. Royal Red Cross, Second Class: Assistant-Matron Gwendolen Lardner, Sister Flora Browning, Sister Mary Blamire-Brown, Sister Evaline Jones, Sister Mary Lutwick (who also received the Military Medal), and Staff Nurse Lucy Nullis, Q.A.I.M.N.S.R.; Sister Mary Mathewson, T.F.N.S.; Sister Annie Hayhurst, Canadian A.N.S.; Miss Mary Marrow, French Red Cross; Miss Hilda Clarkson, and Miss Mary Thomas, V.A.D. The King conferred the Albert Medal on

**ROUND THE HOSPITALS—***(continued)*.

Miss Alice Batt, V.A.D., whose gallantry on the occasion of the fire breaking out at No. 36 Casualty Clearing Station at Rousbrugge on October 1, 1918, has already been described in these columns. It will be remembered that comment was roused by the record of the duties assigned to this lady in assisting the operating surgeon. She is a fine example of the efficiency attained by the Voluntary Aid nurses of the best type. All the above members of the Nursing Services were subsequently received by Queen Alexandra at Marlborough House.

---

At the Investiture held at Buckingham Palace on July 12, the King personally conferred decorations on the following members of the Nursing Services: Royal Red Cross, First Class: Matron Maude Brasier, Matron Roslyn Carr, Matron Margaret Peiniger, Matron Mary Ashlin Thomas, and Assistant-Matron Mabel Chittock, B.R.C.S.; Sister Evelyn Wilson, Australian A.N.S.; Matron Fanny Wilson, New Zealand A.N.S. Royal Red Cross, Second Class: Sister Marian Burnham, Sister Jean Mair, and Sister Bettie Sear, Q.A.I.M.N.S.R.; Sister Margaret McBride, T.F.N.S.; Sister Elizabeth Campbell, and Sister Fanny Law, B.R.C.S.; Sister Elsie Cooke, Australian A.N.S.; Miss Mary Boyd, Miss Olive Bonham-Carter, the Hon. Dorothy Cross, the Hon. Marjorie Cross, Miss Mary Montgomery, Miss Gertrude Peters, and Miss Edith Tillard, V.A.D. The King conferred the Military Medal on Miss Elsie Cross, Queen Mary's A.A.C. All these ladies were subsequently received at Marlborough House by Queen Alexandra.

---

Queen Alexandra appeared greatly to enjoy her reception at the Roehampton Home Hospital for discharged soldiers, which she formally opened on the 9th inst. Her Majesty was received by a distinguished company which included Dame Swift, Matron-in-Chief of the British Red Cross Society and declared the Hospital open "in memory of my dearest friend Lady Ripon, who worked so hard for the wounded." She also alluded gracefully to the generous gift of £500 contributed towards the hospital by the Abysinnians. One of the disabled men was wheeled up in a bathchair to present a bouquet. The house, which contains fifty patients, has been lent for its present purpose by Mr. and Mrs. Charrington, and it is proposed shortly to build a permanent home for the reception of spinal cases requiring continuous care. A very happy spirit pervades the establishment and the skill of the matron and nursing sisters in maintaining cheerfulness in every ward is beyond praise.

---

Queen Mary's interest in babies has often been manifested, but never more charmingly than when on July 12 she visited Dr. Truby King's Infant Welfare Centre at 29 Trebovir Road, Earl's Court. Her Majesty entered into the details of Dr. Truby King's system with her wonted thoroughness, examined his milk chart in the dispensary, and mastered the latest theories relating to four-hour feeds. The main interest centred in the nurseries, where, we understand, the Queen received a vociferous welcome from the babies, somewhat appeased when a flash-light photograph of her Majesty focussed all eyes upon her. The national importance of Dr. Truby King's mission to infants is brought into renewed prominence by the Royal visit. To lessen the strain of motherhood, as he is teaching nurses to do, is to render an incalculable service to the Empire. We learn that the doctor is shortly leaving for New Zealand, and that Miss Patrick, the matron, who has so ably conducted the Centre, is also returning home. General satisfaction is felt that the work of the Institute is, however, to be carried on and extended at St. Thomas's Hospital.

---

The Birmingham Centre of the College of Nursing had under discussion, at a meeting last Saturday, the question of the suggested appointment of a supervisor of nursing education at King's College for Women. In the result the members decided to approve such an appointment, and to give financial support to enable it to be carried into effect. We await with interest the further development of this proposal. Liverpool, as our columns last week showed, is also bestirring itself to give the education of nurses the dignity of other professions.

---

It is a very popular innovation for nursing sisters to take their part in the triumphal processions of the troops which mark victory. No section of the procession on July 5 received louder acclaims than that of the Army Sisters, many of whom were recognised with delight by old patients among the crowd. On the 19th they will again form a picturesque and stirring feature in the pageant. In the Naval Contingent one hundred members of the Women's Royal Naval Service will be headed by the Director, Dame Katharine Furse, and a party of Queen Alexandra's Royal Naval Nursing Service will be included. The honour of taking part in this historic march is greatly coveted, but it needs some powers of endurance, for the route, extending over six and a half miles, is a long one.

---

We are glad to note that Government expects women to take an active part in the administrative work of the Health Ministry. The Act empowers, and indeed enjoins, the Minister to give equal consideration to the suitability of persons of both sexes in making appointments. Some of the administrative posts are such as could most suitably be filled by women with previous nursing experience under public health authorities, or as almoners in voluntary hospitals. We hope also to find that nurses procure election on the consultative councils to be set up in connection with the Health Ministry. Some of the county superintendents and inspectors of district, factory, school, and other branches of nursing possess unrivalled knowledge of the health conditions prevailing in their area, and of the im-

**ROUND THE HOSPITALS—(continued).**

pediments to be removed. There is a great reservoir of original observation almost untapped among nurses of this type, which would serve to render them valuable members of the health committees.

---

The Secretary of State for India announces that fully-trained nurses are required immediately for temporary service with British troops in India. They must be within twenty-seven and thirty-five years of age. Preference will be given to those who have served with the Q.A.I.M.N.S.R. or the T.F.N.S., but nurses now serving are ineligible. Inquiries should be addressed to the Secretary, Military Dept., India Office, Whitehall, marked "Temporary Nurses" on the top left-hand corner of the envelope, and should not contain original certificates or testimonials.

---

Members of Voluntary Aid Detachments are wanted to take service in the sick bays attached to Queen Mary's Auxiliary Army Corps in France. They will receive from £22 10s. to £30 a year, with increments of £2 10s. at the end of every six months' approved service. They will be entitled to uniform and other allowances answering to those received by V.A.D.s serving in military hospitals in France, with £11 active service allowance. The opportunity will be grateful to many V.A.D. nurses whose zeal was by no means exhausted when peace was signed.

---

The dangers incurred by receiving mentally unsound and senile cases in Nursing Homes are not always grasped by nurses. They were illustrated recently when the Superintendent of the Nightingale Nursing Home in Regent's Park was fined £100 with £10 costs for receiving four lunatics in unlicensed houses. The prosecution was undertaken by the Public Prosecutor at the request of the Board of Control. One of the patients was a married woman of 76, obviously weak-minded; one was a man of 56, mentally feeble; a third was a spinster with religious mania, and a fourth was an old woman of nearly 80, chronically insane. These are such cases as proprietors of nursing homes are constantly entreated to receive by relatives who consider asylum treatment reflects a stigma on themselves. There was no contention that they were not kindly treated and the payment received appears to have been very moderate. But the law devised for the protection of individuals incapable of looking after their own interests cannot be too closely enforced.

---

The Council of Queen Victoria's Jubilee Institute for Nurses, in making their annual report for 1918, state the supply of Queen's nurses is a very serious problem. Part of the shortage is undoubtedly due to the war, but there can be no shutting the eyes to the fact that district nursing is not so lucrative as some other branches of the service, and attracts fewer women than it ought to do. The minimum salary of £40 a year fixed last August by the Council is far too low, and the scale of salaries is to be revised shortly. There are now thirty-eight County Nursing Associations in affiliation with the Institute, and 1,999 Queen's nurses were at work on the first day of this year, but this total includes 563 undertaking duty in connection with the war. The grand total of Queen's nurses, village nurses, and midwives working in connection with the Institute at the close of last year in Great Britain amounted to 4,567. It is anticipated that the present year will be one of great progress and expansion, but for this increased funds are urgently required.

---

The salaries at the Royal Infirmary, Sheffield, having undergone a general rise, stand now as follows: Home sister, £85 to £90; ward sister and out-patient sister, £65 to £70; night sister and theatre sister, £75; masseuse, £75; assistant theatre nurse, £45 to £50; probationers, £13, £18; third and fourth year nurses, £23, £33.

---

The authorities at the Royal Free Hospital in revising the salaries of the nursing staff have wisely laid great stress on the need for providing pensions for all nurses entering the service of the hospital. Dating from the first of this month, a scheme has been decided on whereby all probationers making agreement to serve the hospital for four years will enter upon a policy for an annuity of £25 a year at the age of 50. The premiums will be paid by the hospital, and should the nurse leave at the end of her training the policy will become her property. Should she remain in the service of the hospital, the premiums will still be paid for her on the understanding that she will take out another policy on her own account of at least equal value. This is a highly educative and valuable scheme, worthy of imitation in other institutions. The salaries of the ward sisters in the Royal Free Hospital have been at the same time raised according to years of service up to a maximum of £85, with further increases of pay in the case of higher nursing officials. The scheme is estimated to cost an additional £1,000 a year.

---

A fine ceremony took place in Chester Cathedral on July 3, when 1,250 nurses, mainly members of Voluntary Aid Detachments, with a sprinkling of nursing sisters among them, marched in procession to present a banner "to be preserved in the Cathedral Church as a memorial of the work done in Cheshire for the sick, the wounded, and the dying during the War now happily at an end." The names of the 86 Auxiliary Hospitals in the Cheshire County are inscribed on the banner. Her Grace Katherine, Duchess of Westminster, who is president of the local board of the British Red Cross Society, was present with Countess Grosvenor and a distinguished company. After a beautiful service in the Cathedral, a meeting was held in the Music Hall and Sir Arthur Stanley outlined the work awaiting accomplishment by the Voluntary Aid Detachments.

**ROUND THE HOSPITALS**—*(continued).*

The proposed extra 6s. a ton on coal hits no industry more forcibly than institutions for the sick. Managers are already beginning to count the cost of the summer plenishing of the cellar, and this new blow just as salaries are rising all round is a very heavy one. Supplies of wood and peat, when procurable, should be got in at once to secure summer prices, and an effort should be made to diminish still further in every direction the consumption of coal. A very satisfactory and economical mixture for open grates is coke and wood. If logs are dexterously inserted, with coke built up around them, a lasting and cheerful fire can be kept going in the wards at a minimum cost.

The effect of the high prices of fruit and vegetables has been to banish many seasonal luxuries from the institution table this summer. Even where fruit is available from the hospital garden it seems to be too precious to eat, and is prudently hoarded by the housekeeper to be turned into jam. Margarine still holds the field, for even an ounce of butter per head a week is too dear at 2s. 6d. a pound. Meat is unaccountably tough if less scarce than formerly. Bread becomes once more the staple article of food, and is more than commonly delicious after its war transformation. Every care should be taken to provide that it is well baked and palatable, and that brown as well as white bread is served at every meal. The skill of the housekeeper at the moment is tested by the use she makes of breadstuffs and cakes, of cereals, of relishes, and soft cheese.

The Committee and Hospital Board of the Royal Infirmary, Leicester, are making history in their new decision to send one of the Hospital Sisters to take a year's training in the Social Science Department of King's College for Women, with a view to her appointment as Sister-Tutor at the Infirmary. The Board will pay all her fees for tuition and residence, amounting to one hundred guineas, and the selected candidate, Miss Colson, will enter on her year's course next October. Sister Colson was engaged in teaching before she became a nurse and is thus unusually fitted to learn her new duties. It is an interesting departure in many ways. Miss Vincent was strongly influenced in recommending the Board to take this step by the perception that a Sister-Tutor cannot be called into being merely by conferring this title on one of the ward sisters. To teach well a special course of training must be taken, and the exceptional requirements of Sister-Tutors are now understood and sympathetically met at King's College. The proposal to train a Sister-Tutor was strongly supported at the Leicester Infirmary by the Honorary Medical Staff.

The Weir Hospital at Balham has closed down and is now in course of retransformation into a civil hospital. It will be remembered that this beautifully-equipped little institution with thirty beds was on the point of beginning its career when war broke out, and was at once placed at the disposition of the military authorities. By the addition of an annexe containing sixty beds, and by surrendering the entire nurses' quarters for the occupation of the wounded, it was found possible to accommodate 160 soldiers. The nurses and V.A.D.'s were housed in hostels adjoining the main building. The annexe is now being removed, and the hospital is undergoing a thorough renovation. The matron, Miss Cubitt, and two of the sisters who have devoted their energies to the military nursing, will resume their duties in the hospital when it is re-opened under a civil aspect. There is some hope that its scope may be further enlarged by turning one of the hostels into an annexe for tuberculosis cases.

The Bill to grant army rank for nurses in the army service of America, in which thousands of nurses throughout the country are interested, has been introduced in the House of Representatives. The measure provides "that the members of the Army Nurse Corps shall have relative rank as follows: the superintendent shall have the relative rank of major; the assistant-superintendents, directors, etc., the relative rank of captain; chief nurses the rank of lieutenant; and nurses the relative rank of second-lieutenant; and as regards medical and sanitary matters and all other work within the lines of their professional duties shall have and shall be regarded as having authority in and about military hospitals next after the medical officers of the army, and shall wear the insignia of the rank of the army to which they correspond."

The number of nursing sisters who volunteered for service in Russia under the Russian Branch of the Red Cross has proved more than sufficient to fill the vacancies, and the list has now been closed. We record this fact with renewed pride in the valour and self-devotion of our Nursing Services. The work promised to be exceptionally arduous, and the conditions to which these volunteers may be exposed can only be guessed at.

The Tipton and Ocker Hill Nursing Association presented a record of unusually good work at their annual meeting, held under the presidency of Mrs. Levitt. There are thirty nurses always at work in the districts covered, and excellent welfare work has been accomplished in school nursing, ophthalmic and tuberculosis nursing, and midwifery, in addition to the general medical and surgical cases. There is a good school for mothers and a training-school for nurses, at which thirty-four pupils entered for the C.M.B. examination, and all passed. There is money in hand, and the Association contemplates shortly opening a maternity home, much needed, as one of the doctors observed, on account of "the disadvantages and awfulness of some of the homes."

